# Incidence and predictive factors of diaphragmatic dysfunction in acute stroke

**DOI:** 10.1186/s12883-020-01664-w

**Published:** 2020-03-05

**Authors:** José Vicente Catalá-Ripoll, José Ángel Monsalve-Naharro, Francisco Hernández-Fernández

**Affiliations:** 1 Department of Anesthesiology and Critical Care Medicine, Complejo Hospitalario Universitario de Albacete, Albacete, Spain; 2 Department of Neurology, Unit of Interventional Neuroradiology, Complejo Hospitalario Universitario de Albacete, Albacete, Spain

**Keywords:** Stroke, Respiratory, insufficiency, Diaphragmatic paralysis, Ultrasound

## Abstract

**Background:**

The most characteristic clinical signs of stroke are motor and/or sensory involvement of one side of the body. Respiratory involvement has also been described, which could be related to diaphragmatic dysfunction contralateral to the brain injury. Our objective is to establish the incidence of diaphragmatic dysfunction in ischaemic stroke and analyse the relationship between this and the main prognostic markers.

**Methods:**

A prospective study of 60 patients with supratentorial ischaemic stroke in the first 48 h. Demographic and clinical factors were recorded. A diaphragmatic ultrasound was performed for the diagnosis of diaphragmatic dysfunction by means of the thickening fraction, during normal breathing and after forced inspiration. Diaphragmatic dysfunction was considered as a thickening fraction lower than 20%. The appearance of respiratory symptoms, clinical outcomes and mortality were recorded for 6 months. A bivariate and multivariate statistical analysis was designed to relate the incidence of respiratory involvement with the diagnosis of diaphragmatic dysfunction and with the main clinical determinants.

**Results:**

An incidence of diaphragmatic dysfunction of 51.7% was observed. 70% (23 cases) of these patients developed symptoms of severe respiratory compromise during follow-up. Independent predictors were diaphragmatic dysfunction in basal respiration (*p* = 0.026), hemiparesis (*p* = 0.002) and female sex (*p* = 0.002). The cut-off point of the thickening fraction with greater sensitivity (75.75%) and specificity (62.9%) was 24% (*p* = 0.003).

**Conclusions:**

There is a high incidence of diaphragmatic dysfunction in patients with supratentorial ischaemic stroke which can be studied by calculating the thickening fraction on ultrasound. Among these patients we have detected a higher incidence of severe respiratory involvement.

## Background

Acute stroke is the third most important cause of death and the most important cause of disability in Western countries [1, 2]. However, in recent years a reduction has been seen both in incidence and mortality due to the improved preventive and therapeutic measures taken [3].

The motor and/or sensory involvement of one side of the body is the predominant clinical sign, though there are also respiratory disorders which for the moment are poorly characterised in the literature. These depend on the location and extent of the neurological injury [4]. They can be attributed to changes in respiratory mechanics due to involvement of the respiratory control centres or weakness of respiratory muscles [4]. They usually manifest as respiratory failure, atelectasis, lung infections or sleep disturbances [5–7]. Diaphragmatic dysfunction is another potential mechanism of respiratory impairment in stroke [8].

The diaphragm is responsible for performing from 60 to 80% of the inspiratory effort [5, 9, 10]. It shows bilateral innervation from the cervical plexus through two phrenic nerves that are formed from C3 to C5 roots (C4 probably to a greater extent) [11, 12]. Involvement of diaphragmatic muscles or the phrenic nerve can cause diaphragmatic dysfunction [13]. This dysfunction ranges from a partial loss of the ability to generate pressure due to muscle weakness to a full loss of diaphragmatic function due to palsy thereof [14]. Unilateral involvement of this muscle is usually paucisymptomatic [15], and may be an underdiagnosed cause of dyspnoea. On the contrary, the bilateral lesion leads to significant dyspnoea at rest, in particular in a lying-down-on-the-back (supine) position [16–18].

Involvement of the diaphragm in ischaemic stroke has been generally described as a palsy contralateral to the lesion in patients with hemiparesis [1, 5, 6]. A bilateral reduction in mobility has been also seen, with no direct diaphragmatic dysfunction, that can contribute to reducing respiratory function in these patients [19, 20]. This diaphragmatic dysfunction could contribute to the appearance of respiratory failure [5] or other complications such as respiratory infections [1].

The ultrasonographic study of the diaphragm has been developed in recent years to evaluate the function of this muscle [21]. This technique provides a non-invasive method, independent of the effort of the patient, and allows images to be viewed in real time [17, 22, 23].

The objective of our study is to establish the incidence of diaphragmatic dysfunction measured by ultrasonography in a number of patients with acute ischaemic stroke. We have also analysed the relationship of this diaphragmatic dysfunction with the appearance of respiratory impairment (RI) and the main prognostic markers of stroke: vascular territory, clinical severity, degree of hemiparesis and administration of reperfusion treatments.

## Methods

A prospective observational study in a cohort of cases diagnosed with acute ischaemic stroke. Patients diagnosed with supratentorial ischaemic stroke in the first 48 h of hospital admission to a stroke unit or critical care unit were selected from the period between 1 November 2017 and 1 May 2018.

The exclusion criteria were: infratentorial ischaemic stroke, patients not collaborating with the diaphragmatic ultrasonography, non-evaluable study of the thickening fraction (TF), use of maintained assisted ventilation in the first 48 h of hospital stay, and patients with a medical history that could interfere with diaphragmatic mobility, such as known previous diaphragmatic dysfunction, history of chest and upper abdominal surgery, previous chest injury and previous hemiparesis leading to significant functional limitation, defined by the mRS scale (Modified Rankin Scale) as 3 or higher.

In our site, patients are evaluated initially by an on-duty neurologist and a baseline computerised tomography (CT) scan is performed, with administration thrombolytic treatment (alteplase) up to 4.5 h after onset, following the recommended guidelines of the Spanish Society of Neurology of 2014 [24]. In the cases where primary or rescue mechanical thrombectomy (MT) is considered, perfusion CT and angioCT of supra-aortic trunks and brain are performed beforehand. Imaging tests were performed in a Philips 64-detector multi-slice CT, Brilance model (*Koninklijk Philips Electronics N.V., Amsterdam, Netherlands*).

The images are analysed by a neuroradiologist, with MT considered in patients with large vessel occlusion (M1, M2, ICA, intracranial or in tandem, A1, P1, basilar) and time from onset of 8 h or less. Those with an indeterminate time from onset (for instance, stroke on awakening) are considered candidates for MT if they show a perfusion deficit consistent with mismatch of 30% or higher, as measured by perfusion CT. Patients undergoing MT are given general anaesthesia and are extubated systematically after the procedure. They are subsequently admitted to the Critical Care Unit and, if there are no incidents, they are referred to the Stroke Unit at 24 h post-MT. The rest of the patients with acute stroke, including those treated with alteplase, are admitted to the Stroke Unit in the first 24–72 h.

Data were collected from the medical records, including demographics, history of the patients: cardiovascular risk factors, functional classification according to the NYHA scale (New York Heart Association) and history of respiratory diseases [chronic obstructive pulmonary disease (COPD) or sleep apnoea-hypopnoea syndrome (SAHS)]. Clinical data were collected: aetiology of the stroke, vascular territory affected, NIHSS (National Institute of Health Stroke Scale), severe sensory aphasia defined as not understanding instructions, degree of hemiparesis (MRC scale, Medical Research Council), application of reperfusion therapies and their success measured by the TICI scale (Thrombolysis in cerebral infarction) or the TIBI scale (Thrombolysis in Brain Ischemia), as applicable.

A follow-up of 6 months after the event was carried out through two telephone calls (at 3 and 6 months). We obtained data on mortality, mRS at 3 months and respiratory involvement, defined as the occurrence of severe dyspnoea (grade 3–4 of the MRC scale), orthopnoea, respiratory infection and difficulty expectorating. To study the prognosis, mRS was dichotomized: good prognosis (score 0–2) and poor prognosis (score 3–6).

Ultrasound images were obtained with a portable Mindray TE7 ultrasound machine (*Mindray, Shenzhen, P. R. China*), with a probe of 12 mHz frequency. Diaphragmatic ultrasonography was performed with the patient placed in a supine position. The ultrasound probe was placed in the axillary midline from T4 and was guided caudally to identify the three lines corresponding to the diaphragm. After they were located, a section was made in mode M and the maximum thickness and minimum thickness of this muscle were measured. Once the measures were obtained, the TF was calculated using the formula described in previous studies: [(inspiratory thickness - expiratory thickness)/expiratory thickness] [16, 21]. Bilateral ultrasonography of the diaphragm was performed.

TF was measured in normal breathing and forced inspiration, and measurements were performed on both hemidiaphragms. For the measurement in forced inspiration, the patient was asked to perform a deep inspiratory effort at maximum capacity, and the fraction was calculated in patients capable of performing the inspiratory effort at maximum capacity. This process was repeated three times with a minute difference and the average TF was obtained. Diaphragmatic dysfunction was considered as a TF of less than 20% [16, 21, 25].

A descriptive study of the variables was performed by calculating the central tendency and dispersion measures for the quantitative variables and the exact calculation and the percentage for the qualitative variables. The comparison between patients with diaphragmatic dysfunction and no diaphragmatic dysfunction was performed using the Wilcoxon signed rank test. The comparison of the categorical variables between two or more subgroups was performed using the Pearson’s chi-squared test or, if appropriate, Fisher’s exact test. The independent effect of the clinical variables was calculated using a binary multivariate logistic regression model, considering as a dependent variable the overall incidence of respiratory involvement. The Hosmer-Lemeshow goodness-of-fit test was used to assess the overall fit of the model. The variables included in the multivariate analyses, in addition to age, the administration of reperfusion treatments and COPD, were those with statistical significance at *p* ≤ 0.05 in the bivariate models. Odds ratios (OR) were calculated for each variable. To analyse the cut-off point with the best discriminative value of the quantitative variables, ROC (Receiver Operating Characteristic) curve analysis was conducted. A value of *p* ≤ 0.05 was considered significant throughout the study. All the results were analysed with the IBM SPSS Statistics® software Version 19 (*SPSS, Chicago, IL, US*).

### Ethics

It is an observational, non-interventional study, the ultrasonographic data obtained were not used for modification of the standard treatment regimens. The authors of the research asked all patients to sign an informed consent form prior to undergoing diaphragmatic ultrasonography. Good clinical practice guidelines and the declaration of Helsinki were followed. Confidentiality of the data obtained was kept and they were only used for the study purposes. Our clinical research was reviewed and approved by the Clinical Research Ethics committee of the University Hospital Complex of Albacete, and was approved on 17 October 2017 with act number 09/2017.

## Results

During the study period a total of 160 patients with a diagnosis of stroke were admitted, including ischaemic, haemorrhagic and vertebrobasilar stroke. The study subjects were recruited consecutively reaching a total of 72 patients. The final cohort comprises 60 patients, as 12 patients were rejected for not meeting accurately the inclusion criteria (9 cases infratentorial ischaemic stroke, 2 for history of thoracic trauma and 1 patient for a history of liver surgery). The demographic data and the history of the patient are given in Table 1.

**Table 1:** Demographic data and the history of the patient

Age	69.30 years ±9.66 years
Sex	51.7% (*n* = 31) male
Arterial hypertension	63.3% (*n* = 38)
Dyslipidaemia	46.7% (*n* = 28)
Diabetes mellitus	30% (*n* = 18)
Smoking	66.7% (*n* = 40) no smoking
	16.7% (*n* = 10) former smoker
COPD	6.7% (*n* = 4)
SAHS	10% (n = 6)
Dyspnoea	84.7% (*n* = 50) Grade 0
	10.2% (*n* = 6) Grade 1
NYHA scale	60% (*n* = 36) Class 1
	35% (*n* = 21) Class 2
Vascular territory affected	Small vessel 19 (31.7%)
	ACA 4 (6.66%)
	MCA 32 (53.33%)
	PCA 4 (6.66%)
	Carotid 1 (1.66%)
NIHSS	3 (2–6)
Reperfusion therapies	58.3% (*n* = 35) No therapy
	20% (*n* = 12) Thrombolysis
	18.3% (*n* = 11) Thrombectomy
	3.3% (*n* = 2) Both therapies

*ACA* Anterior cerebral artery, *COPD* Chronic Obstructive Pulmonary Disease, *MCA* Middle cerebral artery, *NIHSS* National institute of Health Stroke Scale, *NYHA* New York Heart Association, *PCA* Posterior cerebral artery, *SAHS* Sleep Apnoea-Hypopnoea Syndrome

**Fig. 1:**
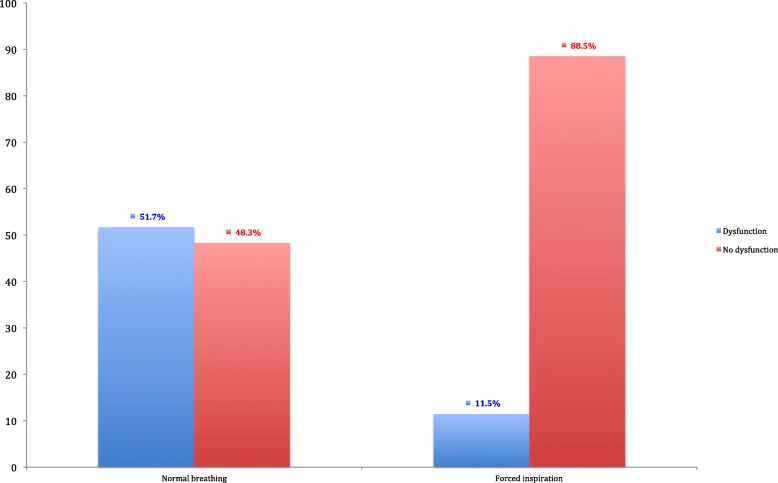
Comparison between dysfunction under normal breathing and forced breathing on the contralateral side. A higher diaphragmatic involvement is seen on the side contralateral to the lesion under normal breathing than when the patient is asked for forced breathing

The overall incidence of diaphragmatic dysfunction in acute ischaemic stroke in the first 48 h following admission was 51.7%. An incidence of diaphragmatic dysfunction of 51.7% was seen under normal breathing and of 11.5% under forced breathing, with a statistically significant difference (*p* <  0.0001). Figure 1 details the differences between basal breathing and forced breathing.

### Diaphragmatic dysfunction under basal breathing

Diaphragmatic dysfunction was seen on the side contralateral to the lesion in 51.7% (*n* = 31) vs 1.7% (*n* = 1) on the ipsilateral side (*p* <  0.0001). The median TF was 19% [12–43%] on the contralateral side vs 37% [33–51%] on the ipsilateral side (p <  0.0001).

### Diaphragmatic dysfunction under forced breathing

Diaphragmatic dysfunction was seen on the side contralateral to the lesion in 11.5% (*n* = 6), while on the ipsilateral side no dysfunction was seen in any patient (*p* = 0.014). The median TF was 38% [27–72%] on the contralateral side vs 75% [55–85%] on the ipsilateral side (*p* <  0.0001).

Patients with diaphragmatic dysfunction had a higher degree of hemiparesis and a higher score on the NIHSS scale, in particular those with a NIHSS score ≥ 6 (68% vs 24%, *p* = 0.023). With regard to the vascular territory involved, 62.5% of the patients with stroke due to occlusion of the middle cerebral artery had diaphragmatic dysfunction (Table 2).

**Table 2:** Diaphragmatic dysfunction in normal and forced breathing according to the clinical examination

	Normal breathing (*n* = 60)		Forced breathing (*n* = 52)	
	Dysfunction	No dysfunction	Dysfunction	No dysfunction
NIHSS				
NIHSS 0–6	14 (40%)	21 (60%)	2 (6%)	31 (94%)
NIHSS 7–15	6 (50%)	6 (50%)	3 (30%)	7 (70%)
NIHSS ≥16	11 (85%)	2 (15%)	1 (11%)	8 (89%)
Hemiparesis degree in the MRC scale				
0	0	0	0	0
1	6 (100%)	0	1 (50%)	1 (50%)
2	6 (100%)	0	3 (50%)	3 (50%)
3	10 (100%)	0	2 (20%)	8 (80%)
4	6 (54.5%)	5 (45.5%)	0	11 (100%)
5	3 (11.1%)	24 (88.8%)	0	23 (100%)

The table shows the relationship between diaphragmatic dysfunction and the NIHSS score and the degree of hemiparesis according to the MRC scale of muscle strength

*MRC* Medical Research Council, *NIHSS* National Institute of Health Stroke

On the other hand, there was no statistically significant difference between diaphragmatic dysfunction and the reperfusion treatment performed (Table 3).

**Table 3:** Reperfusion techniques and diaphragmatic dysfunction

	Normal breathing (n = 60)		Forced breathing (*n* = 52)	
	Dysfunction	No dysfunction	Dysfunction	No dysfunction
No therapy	17 (48.6%)	18 (51.4%)	3 (9.7%)	28 (90.3%)
Thrombolysis	6 (50%)	6 (50%)	2 (20%)	8 (80%)
TIBI 2	2 (100%)	0	1 (100%)	0
TIBI 3	1 (25%)	3 (75%)	1 (25%)	3 (75%)
TIBI 4	1 (100%)	0	0	0
TIBI 5	2 (40%)	3 (60%)	0	5 (100%)
Thrombectomy	8 (73%)	3 (17%)	1 (11%)	8 (89%)
TICI 2b	2 (66.7%)	1 (33.3%)	1 (50%)	1 (50%)
TICI 3	6 (60%)	4 (40%)	0	9 (100%)
Dual therapy	0	2 (100%)	0	2 (100%)

*TIBI* Thrombolysis in Brain Ischaemia, *TICI* Thrombolysis in Cerebral Infarction.

Diaphragmatic dysfunction in basal breathing in the side contralateral to the brain injury was associated with RI (severe dyspnoea, orthopnoea, respiratory infection or difficulty expectorating) in the first 6 months after the stroke, with statistically significant differences to the patients with normal TF (70% vs 30%, *p* = 0.002) (Table 4).

**Table 4:** Respiratory impairment and diaphragmatic dysfunction

	Normal breathing* (*n* = 60)		Forced breathing** (*n* = 52)	
	Dysfunction	No dysfunction	Dysfunction	No dysfunction
No respiratory impairment	8 (30%)	19 (70%)	1 (4%)	25 (96%)
Respiratory impairment - Dyspnoea (*n* = 21, 35%) - Orthopnoea (*n* = 7, 11%) - Respiratory infection (*n* = 14, 23%) - DE (*n* = 22, 36%)	23 (70%)	10 (30%)	5 (19%)	21 (81%)

The table shows the association between diaphragmatic dysfunction and respiratory impairment. The last file shows the incidence of appearance of each symptom during the 6 months of follow-up *p = 0.002. **p = 0.191

*DE* Difficulty expectorating

This association was observed in the bivariate analysis with the TF reduction both under normal (*p* = 0.006) and forced breathing (*p* = 0.003), and also with the female sex (*p* = 0.011), smoking (*p* = 0.028), cardioembolic origin (*p* = 0.025), hemiparesis degree (*p* <  0.001) or the score of the degree in the NIHSS scale (*p* = 0.001) (Table 5).

**Table 5:** Respiratory impairment and characteristics of the patients

	Disorder n (%)	No disorder n (%)	p
Female sex	12 (38.7)	19 (61.3)	**0.011**
Hypertension	21 (55.3)	17 (44.7)	0.957
Dyslipidaemia	16 (57.1)	15 (46.9)	0.755
Diabetes	12 (66.7)	6 (33.3)	0.234
Smoking	7 (35)	13 (65)	**0.028**
Poor performance status	13 (54.2)	11 (45.8)	0.916
Previous severe dyspnoea	3 (100)	0 (0)	0.245
COPD	3 (75)	1 (25)	0.620
SAHS	1 (16.7)	5 (83.3)	0.081
Previous severe hemiparesis	1 (100)	0 (0)	1
Cardioembolic origin	19 (45.2)	23 (54.8)	**0.025**
Sensory aphasia	5 (100)	0 (0)	0.058
Reperfusion treatment	15 (60)	10 (40)	0.511
Hemiparesis, severe	12 (100)	0 (0)	**< 0.001**
Age, mean ± SD	69.5 ± 10.5	69.1 ± 8.8	0.872
NIHSS, median [25%:75%]	9 [3.5:18.5]	4 [2:6]	**0.001**
TF basal breathing %, mean ± SD	21.1 ± 17.2	34.3 ± 18.8	**0.006**
TF forced inspiration %, mean ± SD	37.9 ± 21	57.9 ± 25.3	**0.003**

The table shows the association between respiratory impairment and the demographic and clinical characteristics of the patients. The statistically significant results are shown in bold

*COPD* Chronic Obstructive Pulmonary Disease, *IQ* Interquartile range, *NIHSS* National Institutes of Health Stroke Scale, *SAHS* Sleep Apnoea Hypopnea Syndrome, *SD* Standard desviation, *TF* Thickening fraction

The bivariate analysis relating the characteristics of the patients with mortality at 6 months of study is summarised in Table 6:

**Table 6:** Mortality and mRS and characteristics of the patients

	Mortality			mRS		
	Mortality n (%)	No n (%)	p	Good pr. n (%)	Poor pr. n (%)	p
Female sex	3 (10.3)	26 (89.7)	0.666	19 (65.5)	10 (34.5)	0.855
Hypertension	4 (10.5)	34 (89.5)	0.643	23 (60.5)	15 (39.5)	0.185
Dyslipidaemia	3 (10.7)	25 (89.3)	0.533	17 (60.7)	11 (39.3)	0.360
Diabetes	3 (16.7)	15 (83.3)	0.154	10 (55.6)	8 (44.4)	0.232
Smoking	0	20 (100)	0.099	18 (90)	2 (10)	**0.008**
Poor performance status	3 (12.5)	21 (87.5)	0.380	14 (58.3)	10 (41.7)	0.264
Previous severe dyspnoea	0	3 (100)	1	1 (33.3)	2 (66.7)	0.255
COPD	0	4 (100)	1	2 (50)	2 (50)	0.595
SAHS	0	6 (100)	1	4 (66.7)	2 (33.3)	1
Cardioembolic origin	0	42 (100)	**0.002**	32 (76.2)	10 (23.8)	**0.034**
Sensory aphasia	1 (20)	4 (80)	0.363	0	5 (100)	**0.003**
Reperfusion treatment	3 (12)	22 (82)	0.640	15 (60)	10 (40)	0.412
Severe hemiparesis	1 (2.1)	47 (97.9)	**0.004**	38 (79.2)	10 (20.8)	**< 0.001**
Age, mean ± SD	69.5 ± 10.5	68.9 ± 9.4	0.872	67.2 ± 8.9	73.4 ± 10.1	0.288
NIHSS, median [25%:75%]	21 [18.5:24]	4 [3:9]	**< 0.001**	4 [2:8]	10 [5:20]	**0.006**
TF basal breathing %, mean ± SD	0.14 ± 0.04	0.28 ± 0.19	**< 0.001**	0.31 ± 0.20	0.19 ± 0.13	**0.004**
TF forced inspiration %, mean ± SD	0.40 ± 0.06	0.48 ± 0.25	0.143	0.51 ± 0.26	0.40 ± 0.20	0.061
Mortality	–	–	–	0	5 (100)	**0.003**
RI	5 (8.3)	28 (84.8)	**0.043**	17 (51.7)	16 (48.5)	**0.007**

The table shows the association between mortality and mRS at 3 months and the demographic and clinical characteristics of the patients. The statistically significant results are highlighted in bold

*COPD* Chronic Obstructive Pulmonary Disease, *IQ* Interquartile range, *mRS* Modified Rankin Scale, *NIHSS* National Institutes of Health Stroke Scale, *pr* Prognosis, *RI* Respiratory impairment, *SAHS* Sleep Apnoea-Hypopnoea Syndrome, *SD* Standard deviation, *TF* Thickening fraction

The multivariate analysis evidenced that the degree of hemiparesis (OR = 0.015; 95% CI 0.001–0.227; *p* = 0.002), diaphragmatic dysfunction (OR = 1.095; 95% CI 1.095–1.186; *p* = 0.026) and female sex (OR = 248.312; 95% CI 7.02–8718.144; p = 0.002) were independent predictive factors of RI (Table 7).

**Table 7:** Multivariate analysis of respiratory impairment

Variables	OR	CI = 95% for OR		p
		Upper	Lower	
Age	0.945	0.828	1.079	0.404
Sex	248.312	7.072	8718.144	**0.002**
SAHS	< 0.001			0.999
Cardioembolic stroke	0.049	0.001	2.151	0.118
COPD	2.276 [10]			0.999
Aphasia	1.324 [13]			0.998
Reperfusion treatment	0.142	0.006	3.535	0.234
Post-stroke hemiparesis	0.015	0.001	0.227	**0.002**
NIHSS	0.878	0.692	1.114	0.285
Contralateral TF	1.095	1.011	1.186	**0.026**
Constant	2.183 [8]			**0.004**

The statistically significant results are highlighted in bold

*CI* Confidence interval, *COPD* Chronic Obstructive Pulmonary Disease, *NIHSS* National Institutes of Health Stroke Scale, *OR* Odds Ratio, *SAHS* Sleep Apnoea Hypopnoea Syndrome, *TF* Thickening fraction

**Fig. 2:**
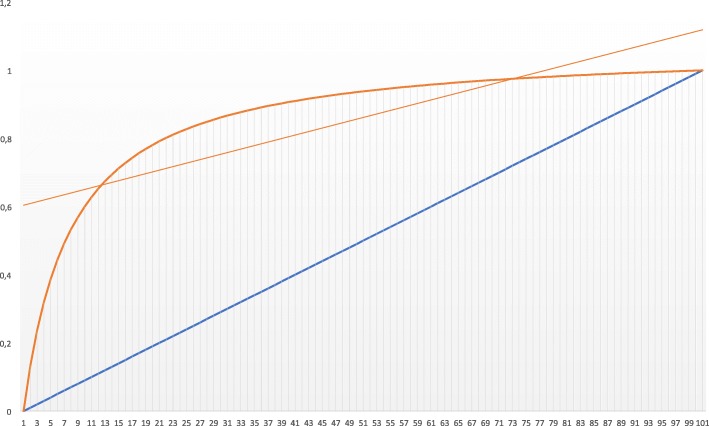
Receiver Operating Characteristic curve: Figure shows the diagnostic accuracy of diaphragmatic ultrasound respect the respiratory impairment. It shows an Area Under Cover of 73% (*p* = 0.003), a sensitivity of 75.7% and a specificity of 62.9%. The graphic has been obtained using Excel 2019 (Microsoft Corporaton Redmond, Washington, United States)

With regard to the diaphragmatic dysfunction shown by ultrasound, the maximum point of maximum diagnostic precision (sensitivity vs specificity) of the TF for the incidence of severe respiratory symptoms is plotted in a ROC curve (Fig. 2), resulting in a cut-off point with a higher AUC (area under the curve) of 24% (AUC 0.73; CI 0.6–0.86, *p* = 0.003), with sensitivity results of 75.7%, specificity of 62.9%, positive predictive value of 71.4%, negative predictive value of 68% and validity index of 70%.

## Discussion

This is the first study to evaluate the incidence of diaphragmatic dysfunction in acute supratentorial stroke, obtaining an overall incidence of 51.7%. This diaphragmatic dysfunction is seen mainly on the side contralateral to the brain injury, where dysfunction is seen in 51.7% of the cases vs 1.7%, where it is seen on the ipsilateral side. Under forced breathing, the increased inspiratory effort reduced the incidence of diaphragmatic dysfunction to 11.5%. The previous studies evaluating diaphragmatic dysfunction in stroke were performed in patients with hemiparesis and none early after the diagnosis [1, 26]. In 1988. Santamaría and Ruiz [27] observed diaphragmatic dysfunction in 62 patients with recent hemiparesis due to supratentorial stroke after comparing the chest X-rays of these patients to those of subjects with brain diseases. Subsequently, in 1994, Cohen et al. [28] reported similar results in patients with hemiparesis between 9 days and 14 months before.

The relationship existing between diaphragmatic dysfunction and ischaemic stroke can be explained both by the change in the voluntary function of the diaphragm due to a lesion in the corticospinal tract and by the lack of automatic control through the bulbospinal tract [5, 6]. However, other studies have suggested that the involvement of the diaphragm in stroke may be due to a disorder in the corticodiaphragmatic pathways [29]. Using magnetic stimulation techniques, it has been seen that the response of the diaphragm to stimulation of a single brain hemisphere is mainly contralateral, and there is a lower response in the ipsilateral side [30]. This response in the ipsilateral side is explained by the presence of small cross connections between the two phrenic nerves, resulting in bilateral innervation of the diaphragm [29]. These connections were also studied in patients with unilateral diaphragmatic dysfunction that show dyspnoea, where an impairment is seen in the pressure generated by the theoretically healthy diaphragm [31].

Diaphragmatic dysfunction is not a disease commonly diagnosed, in particular in the case of an unilateral injury [32]. In our study we used ultrasonography to make the diagnosis of diaphragmatic dysfunction, through the analysis of the TF. The study of lung and diaphragmatic function by ultrasonography was characterised by Khedr et al. in 2000 [5] and Jung et al. in 2014 [6] who observed a correlation between clinical motor deficit in patients with hemiparesis and the hemidiaphragmatic excursion on the side ipsilateral to the hemiparesis. Ultrasonography is a technique with advantages derived from its non-invasive nature, being performed at the bedside of the patient [33] and with no involvement of radiation [22]. In addition, it allows images to be viewed in real time and analyse the muscle independently from the effort of the patient [16, 17, 23]. Using this technique, the diaphragm can be seen as two hyperechogenic layers corresponding to the peritoneal wall and the pleural wall together with an intermediate hypoechoic line corresponding to the muscle itself [16].

Upon breathing, the diaphragm contracts and this results in a muscle thickening proportional to contraction [34]. Therefore, diaphragmatic function can be evaluated by the analysis of thickening of the diaphragm during its contraction, which corresponds to the TF [16]. Although other studies previously measured the diaphragmatic excursion [5, 6], in our case he have studied the TF due to the difficult evaluation of the left window that is obtained with the low-frequency probe necessary for the measurement of excursion [9, 32, 35]. In addition, diaphragmatic excursion reflects the inspiratory volume which, in turn, may be influenced by participation of the accessory muscles, responsible for 20% of the respiratory effort [5, 9, 10].

The sample analysis allowed us to observe that the appearance of diaphragmatic dysfunction occurs mainly in patients with stroke in the territory of the middle cerebral artery, in patients with a score above 6 points on the NIHSS scale (68 vs 24%, *p* = 0.023) and in patients with hemiparesis (*p* = 0.01). It must be highlighted that 23% of the patients with diaphragmatic dysfunction do now show muscle weakness (*p* < 0.001). This may be because corticospinal pathway involvement can be variable and dependent on the stroke territory [36]. With regard to the administration of reperfusion treatments or their result, we have not found any significant relationship with the development of diaphragmatic dysfunction.

The clinical impact of diaphragmatic dysfunction in stroke is still to be elucidated. However, the appearance of respiratory symptoms has been described as a form of presentation of diaphragmatic dysfunction [37]. The TF reduction can be related to a reduction in pulmonary volumes that predispose to respiratory infection [38], as well as a reduction in the inspiratory flow during coughing that limits effective cough [39]. In addition, diaphragmatic dysfunction is related to the appearance of orthopnoea and dyspnoea, frequently underdiagnosed [14]. In our series, 55% of the patients with diaphragmatic dysfunction developed SRI during the the first 6 months of follow-up. The reduction of contralateral TF in basal breathing was also related independently to the RI described (severe dyspnoea, orthopnoea, respiratory infection or difficulty expectorating) (OR = 1.095; 95% CI 1.095–1.186; *p* = 0.026), with a diagnostic precision of 73% (*p* = 0.003, CI 0.6–0.86), and the maximum precision value found was 24%. Other authors have considered a value of 20% as a cut-off point for the diagnosis of diaphragmatic dysfunction [25], as in this study, but we do not know any study that describes a predictive value of respiratory impairment. Other factors related independently to RI were the degree of hemiparesis (OR = 0.015; 95% CI 0.001–0.227; *p* = 0.002) and female sex (OR = 248.312; 95% CI 7.02–8718.144; p = 0.002). It seems logical to consider that a higher degree of hemiparesis will also be associated with a greater diaphragmatic dysfunction, potentially explaining this finding, though we have not measured its severity. The finding of female sex as a predictive factor could be justified by the anatomic variations, including a smaller diaphragm that could account for a reduction of up to 20% in the expansion capacity [40, 41]. The evaluation of the diaphragmatic function after an ischaemic stroke could have potential clinical consequences. Development of respiratory failure, atelectasis and respiratory infections has been described, as well as a reduction in the cough reflex in these patients [37]. In addition, the early diagnosis of this dysfunction could reinforce rehabilitation treatment for the purpose of improving recovery of respiratory function in these patients. Furthermore, we observed a significant relationship between decreased TF and the prognosis, both with mortality and mRS.

Our study sample comprised 60 patients. As it is a pilot study, the sample has been sufficient to obtain the incidence of diaphragmatic dysfunction. However, when dividing the sample into subgroups we found limitations in some comparisons and could not achieve statistical significance, despite noticing a clinical tendency to the differences observed between subgroups. In addition, the limitation for performing deep breathing in some patients has limited the sample number even further in this group. We have not found any study confirming the need to calculate the TF in more than one cycle; however, we have decided to average 3 cycles to avoid possible differences between each of them. A limitation common in many of the studies is the subsequent reproducibility and the within-observer variability that can exist in the same test. It must be noted that within-observer variability, despite being limited in the TF [42], has been removed in our case as all ultrasonographies were performed by the same observer [43].

## Conclusions

The incidence of diaphragmatic dysfunction in patients with supratentorial ischaemic stroke in the first 48 h is high (51.7%). Considering its potential clinical implications, studies are required to evaluate the incidence of respiratory complications in these patients, as well as the impact of respiratory rehabilitation in the clinical prognosis.

## Data Availability

The datasets used and/or analysed during the current study are available from the corresponding author on reasonable request.

## Abbreviations

ACA: Anterior cerebral artery
AUC: Area Under Cover
COPD: Chronic Obstructive Pulmonary Disease
CT: Computerised Tomography
DE: Difficulty expectorating
MCA: Middle cerebral artery
MRC: Medical Research Council
mRS: Modified Rankin Scale
MT: Mechanical Thrombectomy
NIHSS: National Institute of Health Stroke Scale
NYHA: New York Heart Association
OR: Odds Ratio
PCA: Posterior cerebral artery
RI: Respiratory Impairment
ROC: Receiver Operating Characteristic
SAHS: Sleep Apnoea-Hypopnoea Syndrome
TIBI: Thrombolysis in Brain Ischemia
TICI: Thrombolysis in cerebral infarction
TF: Thickening fraction
